# Prevalence of hazardous alcohol use among Spanish primary care providers

**DOI:** 10.1186/s12875-019-0999-3

**Published:** 2019-07-26

**Authors:** Esperanza Romero-Rodríguez, Luis Ángel Pérula de Torres, Juan Manuel Parras Rejano, Fernando Leiva-Cepas, Francisco Camarelles Guillem, Rodrigo Fernández Márquez, José Ángel Fernández García, Collaborative Group Alco-AP, Roger Ruiz Moral, Ana Roldán Villalobos, Sara Fernández López, Pilar Martin-Carrillo Domínguez, Cruz Bartolomé Moreno, Carlos Pérula de Torres

**Affiliations:** 1 Maimonides Biomedical Research Institute of Cordoba (IMIBIC), Reina Sofia University Hospital, University of Cordoba, Cordoba, Spain; 2 Maimonides Biomedical Research Institute of Cordoba (IMIBIC), Reina Sofia University Hospital, University of Cordoba, Teaching Unit of Family and Community Medicine of Cordoba. Program of Preventive Activities and Health Promotion -PAPPS- (semFYC), Cordoba, Spain; 3 Villanueva del Rey Health Center, Andalusian Health Service, Maimonides Biomedical Research Institute of Cordoba (IMIBIC), Reina Sofia University Hospital, University of Cordoba, Cordoba, Spain; 4 Infanta Mercedes Health Center, Madrid Health Service, PAPPS Health Education Group (semFYC), Madrid, Spain; 5 Occidente Health Center, Andalusian Health Service, Cordoba, Spain; 6 Maimonides Biomedical Research Institute of Cordoba (IMIBIC), Reina Sofia University Hospital, University of Cordoba, Villarrubia Health Center, Andalusian Health Service, Cordoba, Spain

**Keywords:** Primary care, Health care professionals, Hazardous drinking, Alcohol, Health system

## Abstract

**Background:**

Alcohol use by health care professionals is one of the potential factors that may affect the prevention of hazardous drinking in Primary Care (PC). The objective of the study was to estimate the prevalence of hazardous alcohol use by PC professionals and assess the existing relationship between socio-demographic and occupational variables of PC professionals and their alcohol use.

**Methods:**

A descriptive, cross-sectional, observational, multicenter study was performed. Location: PC sites of the Spanish National Health Care System (NHS). Participants: Physicians and nurses, who completed an online questionnaire intended to identify the pattern of hazardous alcohol use through the AUDIT-C test. The study population was recruited through random sampling stratified by regions of the PC sites in the NHS. The primary measurements: Frequency of alcohol use, number of drinks containing alcohol on a typical day, frequency of six or more drinks on one occasion.

**Results:**

One thousand seven hundred sixty professionals completed the questionnaire. Hazardous alcohol use was detected in 27.80% (95% CI: 25.5–29.7) of PC providers. The prevalence of hazardous alcohol use was higher in males (34.2%) [95% CI: 30.4–37.6] and professionals aged 56 years or over (34.2%) [95% CI: 28.2–40.2]. The multiple logistic regression analysis revealed a higher hazardous use in males (OR = 1.52; 95% CI: 1.22–1.90), PC physicians (OR = 1.42; 95% CI: 1.01–2.02) and professionals with more time worked (OR = 1.03; 95% CI: 1.01–1.05).

**Conclusion:**

Our study shows the current prevalence of hazardous alcohol use among Spanish PC providers, revealing a higher percentage of hazardous alcohol use in healthcare professionals compared to the Spanish general population. Further interventions are required to increase the awareness of negative consequences derived from alcohol use among PC professionals and its impact on the clinical setting.

## Background

Alcohol use is a major public health problem worldwide. This psychoactive substance is a causal factor in more than 200 diseases and injuries [1]. Its use is associated with the increased risk of health conditions, such as cardiovascular diseases, mental and behavioral disorders, neoplasms, and road traffic injuries. Besides these health consequences, alcohol use has a social and economic impact in the society [2].

According to the latest survey published by the World Health Organization (WHO), the European region has the highest record of per capita alcohol use worldwide (9.8 l) and an increased global disease burden attributable to alcohol [3]. Nationwide, the per capita alcohol consumption identified in Spain was 9.2 l (14.6 in males and 3.8 in females), which is slightly below the European average. The latest Survey on Alcohol and Drugs in Spain (EDADES) [4] reveals that alcohol represents the most commonly consumed legal drug in this country (77.6%), followed by tobacco (40.2%) and hypnosedatives (12%). Based on the previous data, the implementation of preventive activities aimed at reducing alcohol use, conducted by health care professionals, represents a public health priority [5]. Regular visits focused on the approach to hazardous alcohol use or risky alcohol use (level of alcohol use that may be harmful, equivalent to an alcohol intake over 2–2.5 SDUs [Standard Drink Unit = 10 g of pure alcohol]/day in females, and more than 4 SDUs/day in males) [6] may lead to a significant reduction in alcohol consumption in the Primary Care (PC) setting [7].

Currently, multiple factors influencing the development of hazardous alcohol use and the magnitude of the problems related to its intake have been identified in the general population [8]. Among the factors to be considered, alcohol availability, the culture regarding its use, the implementation and compliance with current regulations and the preventive strategies developed by health care professionals should be noted [9]. Among the latter, PC provider’s alcohol use, which may be significantly correlated with their professional alcohol-related practices, should be remarked [10].

Several studies have addressed the level of alcohol use by health care professionals [11–13]. However, no national studies have identified the prevalence of hazardous alcohol use by PC professionals [14–16]. Therefore, the objectives of the present study are: 1) To estimate the prevalence of hazardous alcohol use by PC professionals; 2) To assess the existing relationship between socio-demographic and occupational variables of PC professionals and their alcohol intake.

## Methods

A descriptive, cross-sectional, observational and multicenter study was designed. The study population was formed by health care professionals of PC sites in the Spanish National Health Care System (NHS). The project was conducted from August 2014 to August 2016.

The information was obtained from a questionnaire designed by members of the Family and Community Medicine Teaching Unit of Cordoba, under the expert guidance of the Program for Preventive Activities and Health Promotion (PAPPS, Programa de Actividades Preventivas y de Promoción de la Salud, which belongs to the Spanish Society of Family and Community Medicine -semFYC-, Sociedad Española de Medicina Familiar y Comunitaria) [17]. This questionnaire was created to be self-completed anonymously by each professional, upon the signing of the informed consent and was subjected to a process of consensus, apparent logic, and content validation.

The inclusion criteria were: To be a PC professional (family physician, nurse or family and community medicine resident) of the NHS, and to give consent to participate in the study.

The study population was recruited in several ways:

1. Through the professionals participating in a previous study, the CECC-AP [18], who were recruited through the PAPPS and the Communication and Health Group of the semFYC.
2. By emailing members of semFYC and the Spanish Society of Primary Care Physicians (SEMERGEN, Sociedad Española de Médicos de Atención Primaria).
3. Through random stratified sampling of the PC sites in the NHS, which was performed according to the number of existing centers in each Autonomous Community. An e-mail was sent to the director of the PC site, explaining the objective of the study and encouraging him/her to disseminate the study and to promote survey completion among team members, using the snowball method.

The site random sample was obtained from the catalog of the Ministry of Health [19]. According to this nationwide database, the number of physicians who worked in public PC was 33,482. Assuming that 75% of the sites selected wanted to collaborate in the study, and, from these sites, 4 professionals per PC site and 2 per local clinic on average wanted to collaborate, a sample of at least 430 PC sites and local clinics was considered necessary.

The sample size calculation was determined based on an alpha error of 5%, a precision of 3% and an expected prevalence of alcohol use of 50% (*p* = q = 0.5; maximum indetermination situation), and it was necessary to enroll at least 1,068 professionals in the study.

The overall response rate, considering the membership in scientific societies, was 6.4%. The survey was submitted to 16,474 members of semFYC and 8,000 affiliates of SEMERGEN. Finally, 1,110 members of semFYC and 469 affiliates of SEMERGEN completed the questionnaire.

The study variables analyzed were socio-demographic characteristics (age, sex), occupational characteristics (type of profession, resident trainer, time worked, membership to scientific societies, affiliation to the PAPPS) and hazardous drinking.

The primary outcome of the study was hazardous alcohol use, and it was measured through the AUDIT-C questionnaire [20], abbreviated version of the Alcohol Use Disorders Identification Test -AUDIT- endorsed by the WHO [21]. The AUDIT-C questionnaire consists of three items (frequency of consumption of alcoholic beverages, number of drinks containing alcohol on a typical day, frequency of consumption of six or more SDUs on one occasion), and each item is rated from 0 to 4, with a total potential score that ranges from 0 to 12. Following the criteria established by PAPPS and WHO, a total AUDIT-C score of ≥5 in males or ≥ 4 in females was considered as possible hazardous alcohol use [22].

Surveys were completed online through Google Drive. Data were statistically processed with SPSS v. 17.0 and EPIDAT 3.1 programs. Descriptive statistics were performed and 95% confidence intervals (95% CI) were calculated for the primary study estimators. Subsequently, a bivariate analysis was conducted to verify the relationship of the independent variables and the AUDIT-C questionnaire (Chi-square test, mean comparison test, such as the Student’s T-test or ANOVA test, upon normality check –Kolmogorov-Smirnov test–, using bilateral testing, and *p* ≤ 0.05).

Finally, a multiple logistic regression analysis was conducted to check which variables were associated with hazardous alcohol use. The dependent variable was hazardous drinking (dichotomous variable, obtained from the sum of the three items that form the AUDIT-C questionnaire: Frequency of alcohol use, number of drinks containing alcohol on a typical day, frequency of six or more SDUs on one occasion). The independent variables included in the regression model were: age, sex, type of professional (treated as dummy variable), time worked in PC and resident trainer. Those variables whose *p*-value with Wald test was > 0.05 were deleted, obtaining the most parsimonious model. In order to verify the goodness of fit of the model, the Hosmer-Lemeshow test was used. The project obtained the approval of the Ethics Committee of Hospital Reina Sofía of Cordoba.

## Results

A total of 1,760 PC professionals (75.6% family doctors, 12.5% nurses, 11.4% family medicine residents) participated in the study. 62.9% (95% CI: 60.6–65.2) of the surveyed population were women. The average age of the participants was 47.7 years (SD 11.24; limits: 26 to 64 years; 95% CI: 47.17–48.22), and the mean time worked was 14.10 years (SD 10.55; limits: 1–39; 95% CI: 13.60–14.59) (Table 1).

**Table 1:** Sociodemographic and occupational characteristics of Primary Health Care providers surveyed

Characteristics of providers	n (%)	95% CI
Sex		
Male	653 (37.1)	34.8–39.4
Female	1107 (62.9)	60.6–65.2
Age (years)		
Less than 35	475 (27.2)	24.9–29.1
36–45	432 (24.7)	22.5–26.6
46–55	426 (24.4)	22.2–26.2
56 or more	415 (23.7)	21.6–25.6
Type of provider		
Primary care physician	1330 (75.6)	73.5–77.6
Nurse	220 (12.5)	10.9–14.1
Medical Resident	201 (11.4)	9.9–12.9
Resident Trainer		
Yes	588 (33.4)	31.2–35.6
No	1172 (66.6)	64.4–68.8
Time worked (years)		
Less than 5	486 (27.8)	25.5–29.7
5–10	317 (18.1)	16.2–19.8
11–20	451 (25.8)	23.6–27.7
More than 20	494 (28.3)	26.0–30.2

*95% CI* 95% Confidence Interval

63.5% (95% CI: 61.2–65.7) of professionals surveyed belonged to the scientific society semFYC, 26.8% (95% CI: 24.7–28.9) were affiliated to SEMERGEN, 4.5% (95% CI: 3.5–5.5) were members of SEMG (Spanish Society of General Physicians -Sociedad Española de Médicos Generales-), 1.2% (95% CI: 0.7–1.7) belonged to the nursing society ASANEC (Andalusian Association of Community Nursing-Asociación andaluza de Enfermería Comunitaria-) and 4% (95% CI: 3.1–5.0) were members of other societies not included above. 25.9% (95% CI: 23.8–28.0) of healthcare professionals surveyed were affiliated to the PAPPS.

### Overall, the hazardous drinking recorded in the study population was 27.80% (95% CI: 25.5–29.7)

After analyzing the 3 items of the AUDIT-C questionnaire, 24% (95% CI: 21.5–26.5) of female health care professionals met criteria for hazardous alcohol use, compared to 34.2% (95% CI: 30.4–37.6) of male professionals who had this pattern of alcohol use (Table 2).

**Table 2:** Hazardous alcohol use detected in Primary Health Care providers

Alcohol Use/AUDIT-C	Scores	Female	Male	Total
		n (%)	n (%)	
Frequency of consumption				
Never	0	170 (15.5)	40 (6.2)	210 (12.0)
1–2/ per month	1	318 (28.9)	137(21.1)	455 (26.0)
2–4/ per month	2	390 (73.5)	172 (26.5)	562 (32.2)
2–3/ per week	3	162 (14.7)	151 (23.3)	313 (17.9)
4 or more/ per week	4	59 (5.4)	149 (23.0)	208 (11.9)
Number of drinks containing alcohol on a typical day				
1–2	0	591 (53.8)	206 (31.7)	797 (45.6)
3–4	1	453 (41.2)	374 (57.6)	827 (47.3)
5–6	2	50 (4.5)	63 (9.7)	113 (6.5)
7–9	3	3 (0.3)	6 (0.9)	9 (0.5)
10 or more	4	2 (0.2)	0 (0.0)	2 (0.1)
Drinking 6 or more drinks in one day				
Never	0	907 (82.5)	418 (64.4)	1325 (75.8)
Less than monthly	1	158 (14.4)	183 (28.2)	341 (19.5)
Monthly	2	26 (2.4)	33 (5.1)	59 (3.4)
Weekly	3	6 (0.5)	15 (2.3)	21 (1.2)
Daily	4	2 (0.2)	0 (0)	2 (0.1)
Hazardous drinking (Total AUDIT-C score)		264 (24.0)	222 (34.2)	486 (27.80)

*95% CI* 95% Confidence Interval

Table 3 represents the differences between the group of professionals with risky alcohol use and health care professionals who did not have this pattern of alcohol use, according to socio-demographic and occupational variables. Statistical significance was obtained with regard to age (*p* = 0.005), sex (*p* = 0.001), type of profession (*p* = 0.005), being resident trainer (*p* = 0.060) and time worked (*p* < 0.001).

**Table 3:** Hazardous drinking of Primary Health Care providers according to sociodemographic and occupational characteristics

Variables		No Hazardous drinking n (%)	Hazardous drinking n (%)	*p* value^a^
Age	Less than 35	344 (72.4)	131 (27.6)	0.005
	36–45	329 (76.2)	103 (23.8)	
	46–55	316 (74.2)	110 (25.8)	
	56 or more	273 (65.8)	142 (34.2)	
Sex	Male	427 (65.8)	222 (34.2)	< 0.001
	Female	835 (76.0)	264 (24.0)	
Type of provider	Primary care physician	933 (70.6)	389 (29.4)	0.005
	Medical Resident	158 (76.0)	50 (24.0)	
	Nurse	171 (78.4)	47 (21.6)	
Resident Trainer	Yes	857 (73.6)	307 (26.4)	0.060
	No	405 (69.3)	179 (30.7)	
Time worked	Less than 5	365 (75.1)	121 (24.9)	< 0.001
	5–10	236 (74.4)	81 (25.6)	
	11–20	336 (74.5)	115 (25.5)	
	More than 20	325 (65.8)	169 (34.2)	

^a^ Chi-square Test

According to age, the analysis revealed a higher percentage of hazardous drinking in health care professionals age 56 or older (34.2%; 95% CI: 28.2–40.2), compared to those professionals aged 45–55 years (25.8%; 95% CI: 20.5–31.5) (Table 3). With regard to the participants’ sex, male professionals had a higher percentage of hazardous alcohol use (34.2%; 95% CI: 29.4–39.0). In relation to the type of health care professional, family physicians had the highest percentage of risky alcohol use (29.4%; 95% CI: 26.2–32.7) out of the three professions analyzed.

As shown in Table 4, the variables associated with hazardous alcohol use, through multiple logistic regression analysis and after adjusting the model by age variable, were: Sex (higher in males, OR = 1.52; *p* < 0.001), type of professional (higher risk use among family physicians, compared to nurses, OR = 1.43; *p* = 0.045) and time worked (more likely to have hazardous alcohol use, as the time worked increases, OR = 1.03; *p* = 0.004).

**Table 4:** Variables related to hazardous drinking. Logistic regression final model

Variable	β	OR	95% CI	*p* value
Edad (years)	−0.017	0.98	0.96–1.00	0.087
Sex (Male Vs Female)	0.421	1.52	1.22–1.90	< 0.001
Type of provider (Reference category: nurse)				
-Primary care physician vs. Nurse	0.352	1.42	1.00–2.02	0.048
-Medical Resident vs. Nurse	0.278	1.32	0.81–2.15	0.264
Time worked (years)	0.029	1.03	1.01–1.03	0.005

Dependent variable: Hazardous drinking (Yes vs Not); *OR* Odds Ratio, *95% CI* 95% Confidence Interval; Hosmer-Lemeshow Test: 13.599; *p* = 0.093

## Discussion

This study constitutes the first nationwide analysis focused on the hazardous alcohol use among Spanish PC professionals. The present research is intended to evaluate the current situation of this public health problem and to determine similarities and differences identified in other research studies performed by professionals of several health care settings.

Studies developed in the last decade [23, 24] highlight the role played by health care professionals in the implementation of health promotion and preventive practices aimed at reducing hazardous alcohol use. In this setting, health care professionals’ knowledge, attitude, beliefs and experiences about the recommendations of healthy lifestyles and disease prevention acquire special relevance.

PC professionals’ health behaviors may influence patients’ attitude and motivation to make changes in their lifestyle [25]. One of the potential predictors of health promotion and prevention of hazardous alcohol use is the PC professionals’ personal alcohol use. Several studies reveal a significant association between health providers’ alcohol use and their clinical management to approach this substance in the PC setting, wherein professionals with healthy lifestyles are the most prone to offering preventive advices regarding alcohol [26, 27].

At present, there are local studies focused on the pattern of hazardous alcohol use by PC professionals [14–16]. The percentages of hazardous drinking obtained in these studies are heterogeneous, due to the different criteria used to define risky alcohol use, which is a limitation that should be considered to compare these data with the results obtained in our research.

Rodríguez’s [14] study identifies risky alcohol use in 20.7% female professionals and in 27.7% male professionals. This percentages are lower than those obtained in the present research. Several international studies that approach this issue show a wide variety of results. Rosta [28] detected a risky alcohol use percentage of 19.8% in German health care professionals; this prevalence is similar to that obtained by Joos [29] (18%) in a sample of 1,501 specialists. In addition, Sebo [30] identified a hazardous alcohol use of 31.1% in male professionals and 24% in female professionals, which is a similar percentage to that obtained in our research (34.2% in males, 24% in females).

The prevalence of hazardous alcohol use indicated in this study, compared to published global data of the Spanish population, is higher in both males and females. Additionally, Gual [31] pointed out that the percentage of the Spanish general population who drank over the risk thresholds was 22.1% (32.7% in males and 11.3% in females). This fact significantly contrasts with data obtained in the European Survey of Health in Spain (ESHS) [32] published in 2016, which showed that 1.6% of the population had risky alcohol use (> 40 g/day in males and > 20 g/day in females). One of the main reasons that could explain this contrast is the heterogeneity of criteria used in the diagnosis of risky alcohol use.

The finding of a higher risky alcohol use among PC professionals, compared to the general population, may be explained by the presence of different factors related to the healthcare profession, such as work conditions (number of on-call shifts, work stress, occupational burnout syndrome, or number of work hours per week), degree of professional satisfaction, organizational environment, personal situation (marital status, number of children), or the type of medical specialty [33]. Oreskovich’s [34] study, conducted in a sample of 7,197 surgeons, identified as predisposing factors of hazardous alcohol use the occurrence of burnout (OR 1.25; 95% CI: 1.06–1.48), depression (OR 1.48; 95% CI: 1.26–1.73), or medical malpractice (OR 1.45; 95% CI: 1.17–1.78). This fact highlights the need to analyze potential factors that are related to the professionals’ risky alcohol use. Further research is required to provide help to those professionals in whom a risky alcohol use is detected, and to promote the care of these professionals by means of specific programs developed with this purpose, such as the Comprehensive Health Care Program for Sick Physicians (PAIME, Programa de Atención Integral al Médico Enfermo), which the Spanish Medical College Organization offers its members [35].

In terms of gender, data obtained in the present research are similar to those obtained in the three previous studies conducted in Spain [14–16], where a greater prevalence of use among males was already revealed. In addition, the results of our study are in accordance with those provided by EDADES [4] and ESHS [31], regarding the Spanish population.

Furthermore, our findings reveal a higher percentage of hazardous alcohol use in PC professionals aged between 55 and 65 years old. This greater proportion of older professionals who have risky alcohol use contrasts with the increase in the last few years of binge drinking (intake pattern of 6 or more SDUs in a short period of time) in young Spanish professionals [36]. The Spanish National Survey of Health (ENSE, Encuesta Nacional de Salud de España) [37] of 2017 noted this finding, identifying the higher percentage of binge drinking in males (19.1%) aged between 25 and 34 years and females aged between 15 and 24 years (9.5%).

In relation to the healthcare profession, our data reveal differences between the three healthcare groups analyzed, indicating a higher percentage of risky alcohol use in PC physicians. There are no nationwide studies that allow us to compare these results in the PC context; therefore, further research is required to verify the existence of these differences in alcohol use among PC providers.

### Strengths and limitations

One of the limitations of this study lies in the variability of the measurement of alcohol use, given the wide disparity in the volume of alcohol registered in the literature and the self-reported providers’ alcohol use, which may have caused an underestimation of the prevalence of hazardous alcohol use among PC providers [38]. Another limitation of the study arises from the difficulty to compare our data with the previous results published on this topic, due to the diversity of criteria established in the definition of risky alcohol use throughout the literature, which hinders the drawing of consistent conclusions about the hazardous drinking of PC professionals.

Similarly, it is necessary to bear in mind the potential screening bias, given the voluntariness of health care professionals to answer the questionnaire, where the most motivated professionals in this matter would be the most prone to responding to it, which may underestimate the true prevalence of risky alcohol use. Furthermore, it should be considered that the results of hazardous drinking (positive cases of AUDIT-C) obtained in the present study should be confirmed with the complete AUDIT questionnaire (10 items), following the established criteria by PAPPS and WHO [21].

In order to analyze the representativeness of the sample with respect to the study population, we have compared our data according to age and sex with data published by the Medical College Organization (MCO) [39] in the year 2015. Hence, the proportion of female family physicians in Spain was 54.2%, and this percentage rises to 62.9% in our study; therefore, an overrepresentation of female providers may be deemed. Due to the fact that the prevalence of risky alcohol use among female providers is lower than the prevalence identified among male professionals, we may infer that the overall alcohol intake would be underestimated. In terms of age, a greater proportion of young people is observed among the participants of this study. Considering that a greater percentage of risky alcohol use was observed among the groups of older professionals, this issue may have caused an underestimation of the global prevalence of risky alcohol use. However, the sample of this study represents the Spanish PC professionals, as over 95% of them work in the NHS.

One of the strengths of our study, compared to other studies focused on PC providers’ hazardous drinking, lies in the sample size. Globally, there are studies with a greater sample size [40, 41] although these works address hazardous alcohol use in the hospital setting.

## Conclusions

In conclusion, our study reveals the current situation of hazardous alcohol use of Spanish PC professionals, showing a greater prevalence in this population compared to other international studies published in this area. The present research highlights the need to develop preventive strategies and training interventions focused on the identification of hazardous drinking among health care providers. Further research is required to analyze the PC professionals’ occupational conditions and their lifestyles, including their pattern of alcohol use, in order to provide help to those professionals in whom a hazardous drinking is detected. This fact is highly relevant in the clinical setting, given the key role that health care professionals have in the implementation of preventive practices in the PC setting.

## Data Availability

The datasets used/or analysed during the current study are available from the corresponding author on reasonable request.

## Abbreviations

ASANEC: Andalusian Association of Community Nursing
AUDIT-C: Alcohol Use Disorders Identification Test-C
EDADES: Survey on alcohol and drugs in Spain
ENSE: Spanish National Survey of Health
ESHS: European Survey of Health in Spain
MCO: Medical College Organization
NHS: Spanish National Health Care System
PAIME: Comprehensive Health Care Program for Sick Physicians
PAPPS: Program of Preventive Activities and of Promotion of the Health
PC: Primary care
SDU: Standard drink unit
SEMERGEN: Spanish Society of Primary Care Physicians
semFYC: Spanish Society of Family and Community Medicine
SEMG: Spanish Society of General Physicians
WHO: World Health Organization
